# Diminished carbon and nitrate assimilation drive changes in diatom elemental stoichiometry independent of silicification in an iron-limited assemblage

**DOI:** 10.1038/s43705-022-00136-1

**Published:** 2022-07-09

**Authors:** Michael A. Maniscalco, Mark A. Brzezinski, Robert H. Lampe, Natalie R. Cohen, Heather M. McNair, Kelsey A. Ellis, Matthew Brown, Claire P. Till, Benjamin S. Twining, Kenneth W. Bruland, Adrian Marchetti, Kimberlee Thamatrakoln

**Affiliations:** 1 Marine Science Institute and The Department of Ecology Evolution and Marine Biology, University of California, Santa Barbara, CA USA; 2 Integrative Oceanography Division, Scripps Institution of Oceanography, University of California, San Diego, La Jolla, CA USA; 3 Skidaway Institute of Oceanography, University of Georgia, Savannah, GA USA; 4 University of Rhode Island, Graduate School of Oceanography, Narragansett, RI USA; 5 Department of Earth, Marine and Environmental Sciences, University of North Carolina at Chapel Hill, Chapel Hill, USA; 6 Flagler College, St. Augustine, FL USA; 7 Chemistry Department, California State Polytechnic University, Humboldt, Arcata, CA USA; 8 Bigelow Laboratory for Ocean Sciences, East Boothbay, ME USA; 9 Department of Ocean Sciences, University of California, Santa Cruz, CA USA; 10 Department of Marine and Coastal Sciences, Rutgers University, New Brunwsick, NJ USA

**Keywords:** Biogeochemistry, Molecular ecology, Microbial biooceanography

## Abstract

In the California Current Ecosystem, upwelled water low in dissolved iron (Fe) can limit phytoplankton growth, altering the elemental stoichiometry of the particulate matter and dissolved macronutrients. Iron-limited diatoms can increase biogenic silica (bSi) content >2-fold relative to that of particulate organic carbon (C) and nitrogen (N), which has implications for carbon export efficiency given the ballasted nature of the silica-based diatom cell wall. Understanding the molecular and physiological drivers of this altered cellular stoichiometry would foster a predictive understanding of how low Fe affects diatom carbon export. In an artificial upwelling experiment, water from 96 m depth was incubated shipboard and left untreated or amended with dissolved Fe or the Fe-binding siderophore desferrioxamine-B (+DFB) to induce Fe-limitation. After 120 h, diatoms dominated the communities in all treatments and displayed hallmark signatures of Fe-limitation in the +DFB treatment, including elevated particulate Si:C and Si:N ratios. Single-cell, taxon-resolved measurements revealed no increase in bSi content during Fe-limitation despite higher transcript abundance of silicon transporters and silicanin-1. Based on these findings we posit that the observed increase in bSi relative to C and N was primarily due to reductions in C fixation and N assimilation, driven by lower transcript expression of key Fe-dependent genes.

## Introduction

Diatoms play a key role in global biogeochemistry, accounting for ~30% of marine primary production [1]. Through an obligate silicon (Si) requirement for frustule formation, diatoms serve as the critical link between the ocean carbon (C) and Si cycles, and disproportionately contribute to organic carbon export compared to similarly sized non-siliceous cells [1]. In the late spring and summer, phytoplankton productivity in the coastal California Upwelling Zone (CUZ) is fueled by wind-driven upwelling events that transport deep waters to the surface that are rich in both silicic acid (Si(OH)_4_) and nitrate (NO_3_^−^). The concentrations of both Si(OH)_4_ and NO_3_^−^ in these waters often exceed 20 µmol L^−1^ [2]. Along stretches of narrow continental shelf, the deep ferricline and lack of contact with Fe-laden sediment can result in upwelled water deplete in dissolved Fe (dFe) relative to nitrate (>12 µmol L^−1^ NO_3_^−^:1 nmol L^−1^ dFe) that has the potential to push the system into Fe-limitation, similar to open ocean high nutrient, low chlorophyll (HNLC) regions [3].

Fe-limited HNLC regions are considered “hot spots” of diatom silica burial [4] and they can be areas of enhanced carbon export efficiency [5], with the underlying driving mechanism often cited as an increase in the molar ratio of particulate Si to C and nitrogen (N) that results in enhanced mineral ballast and a more efficient biological pump [5]. Alterations in elemental stoichiometry (here Si:C:N) can arise through a variety of changes, including phytoplankton community structure and cellular physiology [2, 6, 7]. For example, Fe-limitation can shift the phytoplankton community away from non-silicifying taxa or toward more heavily silicified diatom species [8]. Interspecific reductions in cell size [9] or changes in cell shape [10] in response to Fe-limitation can impact the overall cellular stoichiometry, given that biogenic silica (bSi) content is more dependent on cell surface area (SA), while particulate organic carbon (POC) and particulate organic nitrogen (PON) are more related to cell volume (V). The remarkable physiological plasticity of diatoms allows cells to alter bSi [11], N [12], and C [6] content somewhat independently in response to environmental conditions altering cellular elemental stoichiometry. During Fe-limitation, some diatom species produce more heavily silicified frustules, which has been hypothesized to be due to slower growth rates given that silicon uptake and silica production, are closely tied to the cell cycle [5, 13–15]. Increased expression of silicon transporters (*SITs*), a conserved family of membrane-bound proteins responsible for Si uptake into the cell [16], under Fe-limitation has been suggested to be a mechanistic driver behind more heavily silicified frustules [17, 18]. Similar to increases in cellular bSi content, altered elemental stoichiometry could also arise due to reductions in C and N in Fe-limited diatoms, as has been reported in both laboratory- [18, 19] and field-based studies [20–22]. This has been attributed to the replacement of high Fe-requiring proteins involved in C and N assimilation with Fe-independent, but less efficient, analogs under low Fe availability such as the substitution of the Fe-containing photosynthetic electron transport chain proteins, ferredoxin and cytochrome c_6_ with flavodoxin [23] and plastocyanin [24]. Similarly, decreased expression and activity of Fe-dependent nitrate and nitrite reductases [25, 26] during periods of low Fe availability contribute to reduced N assimilation [21, 22].

Given the significant contribution of diatoms to sinking flux in the ocean [27], it is important to understand the mechanisms driving changes in diatom elemental stoichiometry and subsequent impacts on the magnitude of organic matter export into the mesopelagic. Bulk measures of bSi, POC and PON have been invaluable in providing insight into the net community response to Fe-limitation. However, recent studies using single diatom cell-based analyses have failed to find a relationship between Fe limitation and increased silica ballast [6, 28] requiring a reassessment of the factors driving increases in Si:C and Si:N under Fe limitation. We previously described the molecular response of a diatom-dominated, phytoplankton community to simulated upwelling and Fe availability in waters overlying a narrow region of the continental shelf of the CUZ [29, 30]. Here, we combine bulk and single-cell elemental measurements of Fe-limited diatoms to assess the contribution of silica production and cellular silica content to observed increases in Si:C and Si:N. Metatranscriptomic analysis of that same community further enabled interrogation of the underlying molecular drivers of the observed response. In addition to transcriptomic indicators of C and N metabolism, we explore the viability of two well-characterized, and highly conserved proteins silicon transporters (*SITs*) responsible for the uptake of dissolved Si, and silicanin-1 (*Sin1*), a diatom-specific protein [31] involved in silica biomineralization [32]—as molecular indicators of silicon metabolism in natural communities.

## Results

### Initial water mass characteristics

**Fig. 1:**
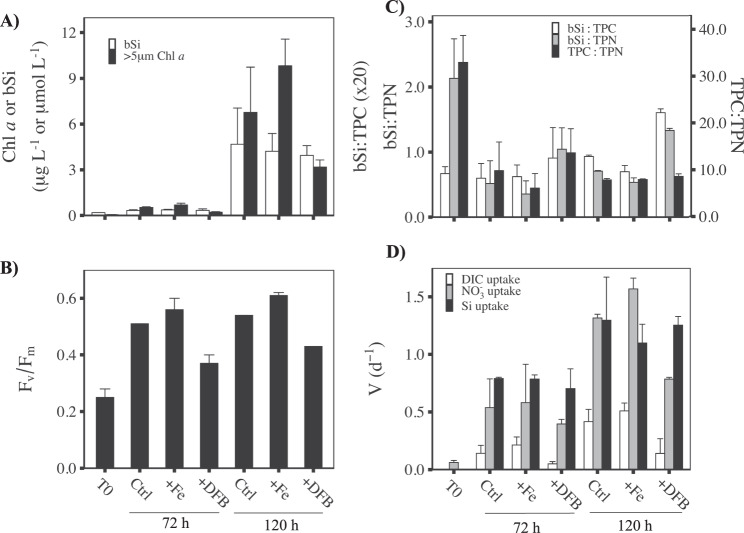
Physiological and biogeochemical characteristics of the initial upwelled water and incubations. Measurements of the initial water mass (T0) and control (Ctrl), iron (+Fe) and DFB (+DFB) additions at 72 and 120 h for (**A**) biogenic silica, bSi (µmol L^−1^; white) and Chl *a* (µg L^−1^; black) concentrations, (**B**) the maximum photochemical quantum efficiency of PSII (F_v_/F_m_), (**C**) molar ratio (µmol L^−1^:µmol L^−1^) of bSi to total particulate carbon (bSi:TPC; white) or nitrogen (bSi:TPN; gray), and TPC:TPN (black) and (**D**) dissolved inorganic carbon (DIC; d^−1^; white), nitrate (NO_3_^−^) uptake rates (d^−1^; gray), and Si uptake (d^−1^; black). Chl *a*, TPC, and TPN data are for the >5 µm size fraction. Mean and standard deviation are shown (*n* = 3). One-way ANOVA and Tukey’s HSD post hoc test results and significance are shown in Table S5.

To investigate the response of diatoms to upwelling, a trace-metal clean Teflon diaphragm pump and tubing was used to collect seawater from 96 m in the upwelling zone off coastal California, corresponding to the 10 °C isotherm as presented by Lampe et al. [29]. Satellite-derived sea surface temperatures (SST) combined with shipboard measurements of SST, wind velocity, and low surface nitrate + nitrite concentration, here-after referred to as nitrate (NO_3_^−^) concentration, indicated that upwelling-favorable conditions were not present upon arrival at our study site or for the previous 13 days [29], setting the potential upper limit on the length of time phytoplankton cells had spent at depth. Initial (T0) phytoplankton abundance in the experimental water was low as indicated by chlorophyll *a* concentration (Chl *a;* >5 µm), 0.05 µg L^−1^, bSi concentration, 0.19 µmol L^−1^, and diatom cell abundance, 5.8 × 10^3^ cells L^−1^ (Fig. 1A). The initial community also had low photosynthetic efficiency (F_v_/F_m_ = 0.25 ± 0.03) and a total particulate carbon (TPC) and nitrogen (TPN) ratio of 31.5 (>5 µm), ~5-fold higher than the Redfield ratio of 6.6 (Fig. 1B, C, Table S4). High TPC:TPN is not atypical of deep or freshly upwelled waters in the CUZ and likely indicates prior N-limitation [33, 34] and/or a high C-rich detrital fraction.

The deep water was rich in NO_3_^−^ and Si(OH)_4_, with concentrations of both near 22 µmol  L^−1^ (Fig. S1, Table S1). The initial dFe concentration, 0.82 nmol L^−1^, was below the average of ~1 nmol L^−1^ typical of the CUZ [35], but was likely sufficient to support growth of the sparse initial phytoplankton community. However, the high NO_3_^−^:dFe ratio (26 µmol L^−1^ NO_3_^−^: 1 nmol L^−1^ dFe) was more than double the threshold of 12 µmol L^−1^ NO_3_^−^:1 nmol L^−1^ dFe, indicating that nutrient utilization and biomass accumulation would lead to eventual Fe limitation [3].

### Bloom progression under varied iron availability

Seawater was subsampled into incubation bottles and left unamended (Ctrl), augmented with either 5 nmol L^−1^ FeCl_3_ (+Fe) to relieve potential Fe limitation, or 200 nmol L^−1^ of the siderophore desferroxamine-B (+DFB) to induce Fe limitation. Bottles were incubated in flow-through surface seawater cooled incubators (12–18 °C) screened to 33% incident irradiance. After 72 h, the phytoplankton assemblage responded positively on a photosynthetic level to the simulated upwelling in all of the treatments, with F_v_/F_m_ increasing markedly from 0.25 ± 0.03 at T0 to 0.51 ± 0.00, 0.56 ± 0.04, and 0.37 ± 0.03 in the Ctrl, +Fe, and +DFB treatments, respectively, indicating a recovery of photosynthetic efficiency. The addition of DFB limited the increase of F_v_/F_m_ to ~70% of that measured in the Ctrl and +Fe treatments (Fig. 1B).

Biomass (Chl *a*, bSi, TPC, and TPN) started to increase by 72 h and became significantly higher by 120 h in all treatments compared to T0 (Fig. 1A, Table S2). By 120 h, Chl *a* concentration increased in the Ctrl and +Fe treatments to 6.76 ± 2.97 and 9.82 ± 1.75 µg L^−1^, respectively, but was significantly lower (3.18 ± 0.47 µg L^−1^) in the +DFB treatment compared to the +Fe treatment. There was a similar increase in bSi by 120 h to 3.95–4.68 µmol L^−1^, but this did not differ significantly between treatments (ANOVA, *p* = 0.85; Fig. 1A). Finally, the concentrations of TPC (24.6 ± 3.81 µmol L^−1^) and TPN (2.97 ± 0.51 µmol L^−1^) within the +DFB treatment remained ~50% below the +Fe treatment at 120 h, but this fell just below the threshold of statistical significance (Tukey’s HSD; *p* = 0.065 and *p* = 0.068, respectively; Table S5).

The addition of Fe resulted in a significant increase in the biomass-specific NO_3_^-^ uptake rate by 120 h, 1.57 ± 0.09 d^−1^, which was 19% higher than the Ctrl, 1.32 ± 0.03 d^−1^ (Tukey’s HSD; *p* = 0.01), and ~70% higher than the +DFB treatment, 0.79 ± 0.02 (Tukey’s HSD; *p* < 0.001; Fig. 1D, Tables S3, S5). The dissolved inorganic carbon (DIC) uptake also increased ~75% at both 72 h, 0.21 ± 0.07 d^−1^, and 120 h, 0.51 ± 0.07 d^−1^in the +Fe treatment compared to +DFB (0.05 ± 0.02 d^−1^ and 0.14 ± 0.13 d^−1^ at 72 and 120 h, respectively; Fig. 1D, Table S3). In contrast, the Si uptake rates at 72 h, 0.76 ± 0.09 d^−1^, and 120 h, 1.22 ± 0.23 d^−1^ did not differ significantly between Ctrl, +Fe, or +DFB treatments.

The increased concentrations of bSi, TPC, and TPN across various treatments resulted in significant differences in the cellular elemental stoichiometry. The initial TPC:TPN of ~30 decreased to Redfield proportions [36] in all treatments (Ctrl, 7.6 ± 0.3, +Fe, 7.7 ± 0.1, and +DFB, 8.3 ± 0.5) by 120 h (Fig. 1C). Both bSi:TPC and bSi:TPN increased in the +DFB treatment by 120 h (0.08 ± 0.003 and 1.33 ± 0.03, respectively) compared to the +Fe treatment (0.03 ± 0.004 and 0.54 ± 0.07, respectively; Fig. 1C, Table S4). Despite substantial biomass accumulation within the incubations, Si(OH)_4_ and NO_3_^−^ concentrations both remained >14 µmol L^−1^ (Fig. S1), which is not considered rate limiting in the CUZ [37].

**Fig. 2:**
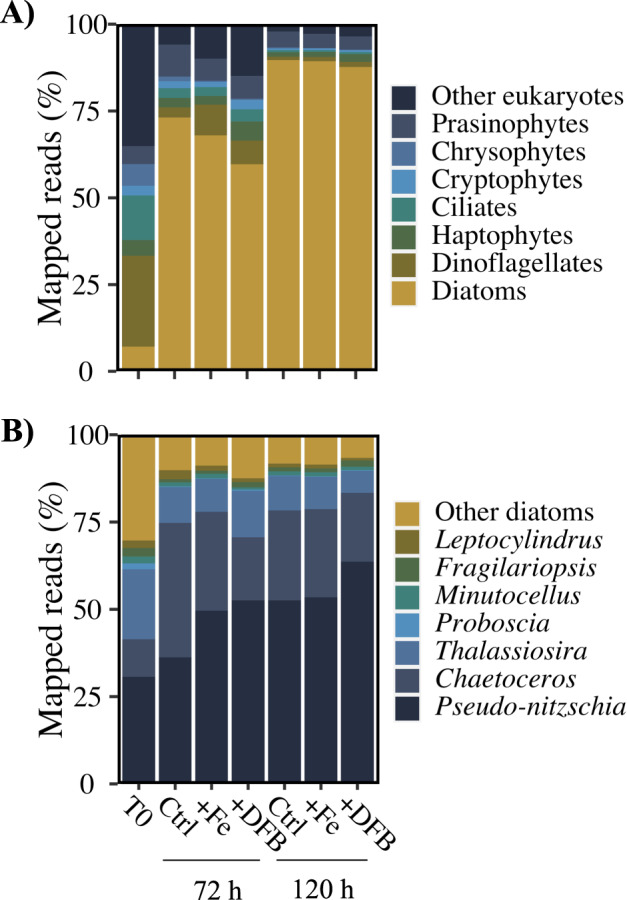
Community composition in initial water and throughout the incubation. Percentage of mapped transcriptomic reads belonging to each of the (**A**) major eukaryotic phytoplankton, and (**B**) diatom genera. Data shown from 120 h are the averages of replicates (*n* = 3).

To assess changes in phytoplankton community composition, we used metatranscriptome sequence data to quantify the relative proportion of taxonomically annotated mRNA reads. Previous comparisons to cell enumeration via microscopy established that mRNA reads are a reasonable proxy for the relative abundance of active phytoplankton groups in this system [29]. A substantial fraction of the mRNA reads in the initial phytoplankton community were associated with dinoflagellate (26%) and diatom (7%) taxa, with the remaining 54% divided among 44 unique taxa (Fig. 2A and Table S6). Across all three treatments, there was a shift toward diatoms, which accounted for 60–73% of the sequence reads at 72 h and ~89% at 120 h (Fig. 2A). This is consistent with our previous report that diatom cell abundance in the Ctrl treatment increased from 5.8 ± 2.5 × 10^3^ cells L^−1^ to ~2.5 ± 0.2 × 10^6^ cells L^−1^ [29]. *Thalassiosira* and the bloom-forming diatoms, *Chaetoceros* and *Pseudo-nitzschia*, were the dominant genera at both sampling timepoints, accounting for a combined 83–90% of total diatom reads across all treatments (Fig. 2B). Although qualitative differences in the relative abundance of *Pseudo-nitzschia* between treatments were present at 120 h, the mean contributions of *Pseudo-nitzschia* to total diatom reads in Ctrl, 53 ± 17%, +Fe treatment, 52 ± 8%, and +DFB, 63 ± 5%, was not significantly different (ANOVA, *p* = 0.50; Fig. S2, Table S5). Furthermore, the genus level distribution of mRNA reads did not differ significantly in the overall community or the diatom community at 120 h (PERMANOVA; *p* = 0.40, *p* = 0.45, respectively). The large proportion of diatom reads, dominated by these three genera, provided an opportunity to interrogate genus-specific responses the increase in the bulk ratios of bSi to TPC and TPN at conditions of low dFe availability.

### Molecular response to Fe-limitation

In addition to the aforementioned biogeochemical and physiological indicators of Fe-limitation within the +DFB treatment, all three dominant diatom genera mounted a molecular response to Fe-limitation. Relative to the +Fe treatment, transcripts of Iron Starvation Induced Protein 3 (ISIP3) were elevated in *Thalassiosira* and *Chaetoceros* in the +DFB treatment at both timepoints (Fig. S3A, B; [38]). Additionally, at 120 h *Thalassiosira* ISIP3 transcripts were significantly elevated in the Ctrl treatment compared to the +Fe treatment while *Chaetoceros* ISIP3 transcripts did not significantly differ between Ctrl and +Fe treatments (Fig. S3A, B, Table S5). For *Pseudo-nitzschia* the iron limitation index (*Ps-n* ILI) was >0.5 in the +DFB treatment at both 72 and 120 h, indicative of Fe limitation [39]. Negative *Ps-n* ILI values in Ctrl and +Fe treatments at both timepoints indicated a lack of Fe-limitation (Fig. S3C).

#### Carbon metabolism

Due to the high Fe requirement of the photosynthetic apparatus, Fe-limitation has a notable impact on C assimilation. When comparing the +DFB to the +Fe treatment we observed changes in the abundance of transcripts of genes involved in photosynthetic electron transport consistent with a molecular response to Fe-limitation [18, 20, 40, 41]. Low RNA yield from samples collected at 72 h necessitated pooling of biological replicates prior to sequencing, limiting the ability to resolve statistically significant differences. However, these data revealed important trends that were recapitulated at 120 h.

**Fig. 3:**
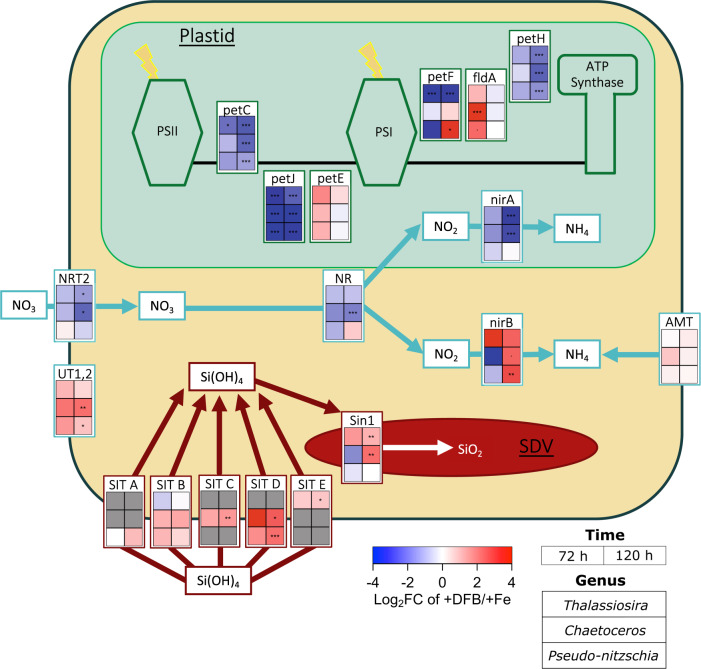
Differential transcript abundance of key cellular carbon, nitrogen, and silicon pathways under Fe limitation for the three dominant diatom genera, *Thalassiosira*, *Chaetoceros*, and *Pseudo-nitzschia*. Heatmap of changes transcript abundance (log_2_ fold-change) in +DFB compared to +Fe treatment at 72 h (left column) and 120 h (right column) for *Thalassiosira* (top row), *Chaetoceros* (middle row), and *Pseudo-nitzschia* (bottom row). Gray boxes indicate genes that were not detected in either treatment. Asterisks denotes false discovery rate (FDR);^.^ *p* < 0.1; *p < 0.05; **p < 0.01; ***p < 0.001. flavodoxin (*fldA*), plastocyanin (*petE*), ferredoxin (*petF*), cytochrome c6 (*petJ*), cytochrome b6/f complex (*petC*), ferredoxin-NADP+ reductase (petH), nitrate reductase (*NR*), nitrate transporter (*NRT2*), ferredoxin-nitrite reductase (*nirA*);, NAD(P)H-nitrite reductase (nirB), ammonium transporter (AMT), urea transporters (*UT1,2*), Silicanin-1 (*Sin1*), *SIT* Clade A (*SIT* A), *SIT* Clade B (*SIT* B), *SIT* Clade C (*SIT* C), *SIT* Clade D (*SIT* D), *SIT* Clade E (*SIT* E).

At 72 h, *Thalassiosira, Chaetoceros*, and *Pseudo-nitzschia* in the +DFB treatment exhibited a >2-fold downward trend in the abundance of transcripts encoding the Fe-dependent electron transport proteins ferredoxin reductase (*petH*), cytochrome b_6_f (*petC*), as well as the Fe-containing ferredoxin protein (*petF*), compared to the +Fe treatment (Fig. 3). At the same time, transcript coding for plastocyanin (*petE)* and flavodoxin (*fldA*), the Fe-independent analogs of *petJ* and *petF* [42, 43], respectively, were generally more abundant in the +DFB treatment (Fig. 3). Notably, transcripts coding for cytochrome c_6_ (*petJ*), were significantly less abundant in all three diatom genera at both 72 and 120 h. By 120 h, genus-specific patterns in *petF* expression emerged with transcripts encoding *petF* within *Thalassiosira* continuing to be significantly less abundant in the +DFB treatment, while *Pseudo-nitzschia petF* transcripts were significantly elevated relative to the +Fe treatment (Fig. 3). In addition, at 120 h all three genera no longer exhibited higher transcript abundance of *fldA* in +DFB compared to +Fe treatment, despite prior significant differential abundance of *fldA* in *Chaetoceros* at 72 h.

#### Nitrogen metabolism

The impact of Fe limitation on N assimilation may also drive increased bSi:TPN characteristic in low Fe regimes [6, 44]. Transcripts for genes encoding ferredoxin-dependent nitrate reductase (*NR*), nitrite reductase (*nirA*), and a nitrate transporter (*NRT2*) were generally less abundant in the +DFB treatment compared to the +Fe treatment for *Thalassiosira* and *Chaetoceros* throughout the incubation. At 120 h, transcripts for all three genes were significantly lower in *Chaetoceros*, while those for *nirA* and *NRT2* were significantly less abundant in *Thalassiosira* (Fig. 3). Transcripts encoding for ferredoxin independent NADH-nitrite reductase protein [45], *nirB*, were >2-fold more abundant in the +DFB treatment relative to +Fe in *Thalassiosira* by 72 h, and in *Chaetoceros* and *Pseudo-nitzschia* by 120 h, though the increase was not significant in *Thalassiosira* at either timepoint (Fig. 3). While the abundance of ammonium transporters (*AMT*) transcripts was unaffected by Fe availability, transcripts encoding urea transporters (*UT*) were generally more abundant in the +DFB treatment in all three diatom genera at both 72 h and 120 h, and significantly more abundant in the +DFB treatment in *Chaetoceros* and *Pseudo-nitzschia* at 120 h. Elevated levels of UT transcripts may have helped diatoms exploit an alternative source of nitrogen that is more efficiently assimilated without the added Fe required for reducing nitrate and nitrite (Fig. 3; [46]).

#### Silicon metabolism

Unlike C and N metabolism, there are no established molecular markers for Si metabolism. The expression of specific clades of diatom *SITs* has been linked to silica production under Fe-limiting conditions [17]. For *Sin1*, expression may be associated with changes in biomineralization that impact the amount of Si deposited per unit area of cell wall [47], referred to here as the degree of silicification. Phylogenetic analysis of *SIT* sequences revealed the presence of genera-specific *SIT* clades (Table S7, [48]), with *Thalassiosira* expressing *SITs* from clades B and E, *Chaetoceros* expressing *SITs* from clades B, C, and D, and *Pseudo-nitzschia* expressing *SITs* from clades A, B, and D (Figs. 3,  S4). In all three genera, respective *SIT* clades C, D, and E transcripts were generally more abundant in the +DFB treatment compared to +Fe at 72 h and and progressed to were significantly higher abundance at 120 h (Fig. 3). Similar qualitative increases at 72 h were observed for clade A and B *SIT* transcripts in *Pseudo-nitzschia*, and clade B transcripts in *Chaetoceros* (Fig. 3). For *Sin1*, transcripts from *Chaetoceros* and *Pseudo-nitzschia* qualitatively decreased at 72 h but increased in *Thalassiosira* in the +DFB compared to +Fe treatment. By 120 h, *Thalassiosira* and *Chaetoceros* had significantly more (>2-fold) *Sin1* transcripts in the +DFB treatment relative to in the +Fe treatment.

### Diatom cell morphology and Si content

The fluorescent dye PDMPO (2-(4-pyridyl)-5-((4-(2-dimethylaminoethylaminocarbamoyl) methoxy) phenyl) oxazole) was used in combination with confocal microscopy to measure single-cell rates of bSi production (Si cell^−1^ d^−1^) and production of new siliceous frustule surface area (SA; µm^2^ cell^−1^ d^−1^) at 72 h when molecular and biogeochemical measures first indicated a differential response to Fe availability. Together, these metrics were used to calculate the degree of silicification (Si µm^−2^) of the frustule (Supplementary Information, Eq. 1; [28]). Single-cell bSi measurements were grouped into four taxonomic classes corresponding to *Chaetoceros*, *Thalassiosira* (consisting of remaining cells with centric morphology), and two morphologically distinct groups of *Pseudo-nitzschia* (45 and 90 µm in apical length). Although PDMPO data is not available from samples at 120 h, the 72 h PDMPO data should be representative of the latter timepoint, as Si uptake was comparable between treatments within each timepoint, macronutrient status remained replete throughout the incubation, and Fe availability was similar within +Fe and +DFB treatments at 72 and 120 h.

**Fig. 4:**
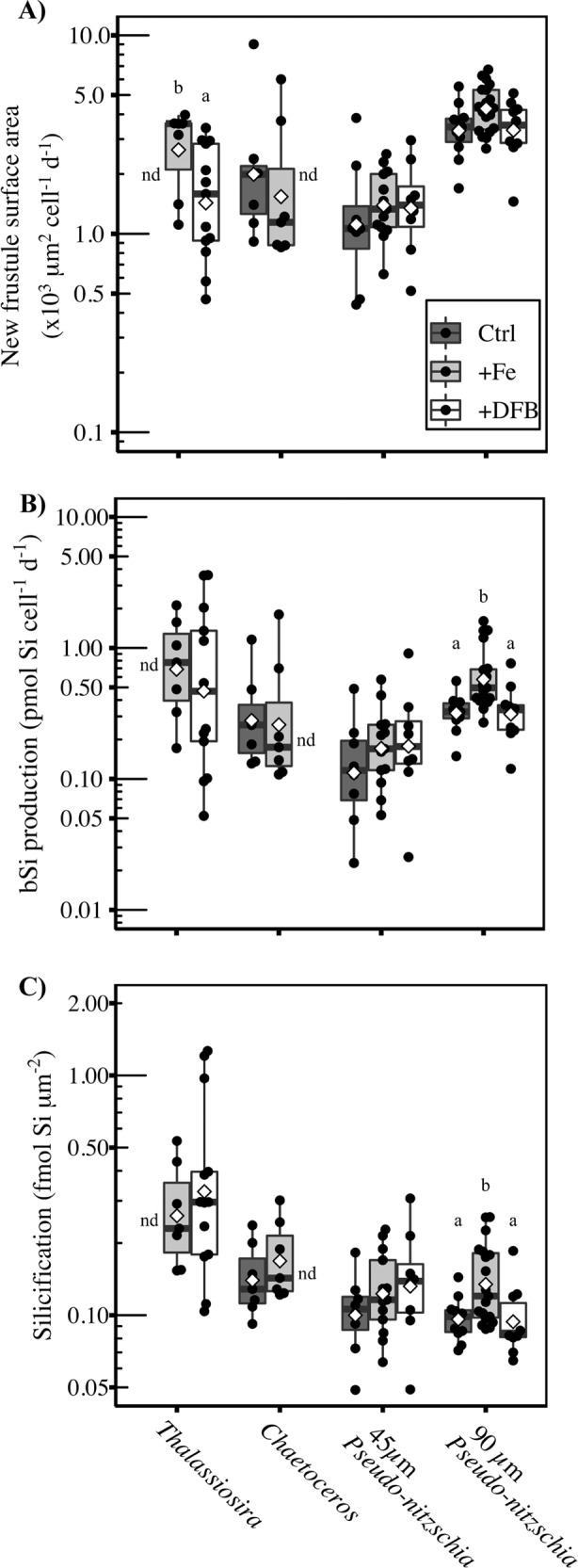
Silicon metabolism-related parameters for diatom taxa obtained using PDMPO. Box plots of (**A**) new frustule surface area (µm^2^ cell^−1^ d^−1^), (**B**) bSi production (pmol Si cell^−1^ d^−1^), and (**C**) degree of silicification (fmol Si µm^−2^) in control (Ctrl; dark gray), +Fe (light gray), and +DFB (white) treatments at 72 h for *Thalassiosira* (all centric diatoms not including *Chaetoceros*), *Chaetoceros*, and *Pseudo-nitzschia* (45 and 90 µm size groups). The center line represents the median and the boxes display the upper and lower quartiles with whiskers extending 1.5 times the interquartile range. Open diamonds show the arithmetic mean of a given sample. Black dots represent data from individual cells. Samples with <5 cells imaged were excluded and denoted nd. Significance was determined within each taxonomic group by Welch two-sample *t* test when comparing between two treatments or one-way ANOVA and Tukey’s HSD post hoc test when comparing among three treatments. Different lowercase letters delineate the statistically significant different groups within a taxa (*p* < 0.05). The vertical axes are plotted on a logarithmic (log_10_) scale. Statistical test results and significance are shown in Table S5.

Despite morphological and physiological differences between *Thalassiosira* (Fig. S5A–C)*, Chaetoceros* (Fig. S5D–F), and *Pseudo-nitzschia* (Fig. S5G–I), there was no difference in the amount of new frustule SA produced by *Chaetoceros* or *Pseudo-nitzschia* in any of the treatments. In *Thalassiosira*, the rate of new frustule SA produced in the +DFB treatment, 1.73 ± 1.03 µm^2^ cell^−1^ d^−1^, was 41% lower (*t* test; *p* = 0.04) compared to the +Fe treatment, 2.92 ± 1.16 µm^2^ cell^−1^ d^−1^ (Fig. 4A). The reduction of the rate of new SA produced by *Thalassiosira* cells within the +DFB treatment compared to the +Fe treatment was accompanied by a significant increase (*t* test; *p* = 0.01) in SA:V (Fig. S6B). These morphological changes are likely associated with an overall reduction in the size of *Thalassiosira* cells because when the proportions of a cell are reduced, V decreases at a faster rate than SA, resulting in an increase in SA:V.

Previously Fe-limitation has been associated with increases in bulk bSi concentration, but we did not observe an increase in cell-specific silica production rates or in the degree of silicification by Fe-limitation (Fig. 4B, C) for *Thalassiosira, Chaetoceros*, or *Pseudo-nitzschia* (Fig. 4B, C). In fact, the >90 µm *Pseudo-nitzschia* cells exhibited a twofold higher silica production rate (Tukey’s HSD; *p* < 0.05), 0.66 ± 0.4 pmol Si cell^−1^ d^−1^, in the +Fe treatment compared to the Ctrl, 0.33 ± 0.10 pmol Si cell^−1^ d^−1^ or the +DFB treatment, 0.35 ± 0.18 pmol Si cell^−1^ d^−1^ (Fig. 4B). This was primarily driven by a 47% increase in the degree of silicification relative to the Ctrl (Tukey’s HSD; *p* = 0.03) and +DFB (Tukey’s HSD; *p* = 0.04) treatments (Fig. 4C). In addition to increasing the cell-specific silica production rate and degree of silicification, Fe addition also increased the mean length-normalized-width (LNW) of the larger *Pseudo-nitzschia* cells by 22% relative to +DFB (Tukey’s HSD; *p* = 0.001; Fig. S6A). This modest increase in the LNW of >90 µm *Pseudo-nitzschia* cells did not, however, result in an increase in SA:V (Fig. S6B).

## Discussion

### Fe limitation led to changes in cellular stoichiometric ratios

In the unamended Ctrl treatment, high initial NO_3_^−^:dFe (26 μmol L^−1^ NO_3_^−^ :1 nmol L^−1^ dFe) indicated that nutrient consumption had the potential to drive the phytoplankton community into severe Fe-limitation [5, 49]. Yet, over the 5-day incubation <30% of the initial nitrate was consumed and the unamended phytoplankton community only exhibited signs of mild Fe-limitation characterized by reduced photosynthetic efficiency and elevated molecular markers of Fe limitation compared to the +Fe treatment. The addition of DFB led to a distinct physiological and molecular response with photosynthetic efficiency, ISIP3 expression (for *Thalassiosira* and *Chaetoceros*) and *Pn*-ILI (for *Pseudo-nitzschia*) illustrative of Fe-limitation in this community.

Iron availability affected uptake rates and ultimately altered bulk elemental composition. Iron-limited communities were also characterized by diminished biomass-specific DIC and NO_3_^−^ uptake rates. In contrast, biomass-specific Si(OH)_4_ uptake rates and bSi concentrations remained similar between Fe treatments. Notably, Fe-limitation negatively impacted both C and N assimilation to a similar extent, such that the particulate C:N ratio was near Redfield proportions at the end (*t* = 120 h) of the incubation [36]. Collectively, these responses resulted in elevated ratios of Si:C and Si:N under Fe-limitation. Previous field campaigns in the CUZ have reported similar increases, as well as increased consumption of Si(OH)_4_ relative to NO_3_^−^ [2, 13, 50, 51], but the drivers of these shifting elemental ratios are often ambiguous. By combining measurements of bulk bSi with single-cell silica production, single-cell degree of silicification and metatranscriptomics, we were able to explore two possible over-arching drivers of this cellular elemental shift.

### Increased SA:V as a driver of elevated Si:C and Si:N

Elevated cellular Si:C and Si:N can be driven by an increase in the total SA of silicified cells relative to the total V of cells in the phytoplankton community. One possible explanation for an increase in SA:V would be intraspecific reductions in diatom cell size [9, 52] or changes in cell shape such as LNW [10]. Given that the diatom cell is encased in a siliceous frustule and that biovolume is associated with C and N rich internal components, a shift toward species with a higher SA:V has the potential to increase Si:C and Si:N without necessarily increasing the degree of frustule silicification [53]. Our data demonstrate that Fe-limitation did not alter SA:V in the most abundant genera *Chaetoceros*, and *Pseudo-nitzschia* despite a reduction in LNW of the large size class of *Pseudo-nitzschia* in response to Fe-limitation. While cell SA:V increased within the *Thalassiosira* genera, the binning of all non-*Chaetoceros* centrics into this classification may have been a contributing factor.

Concomitant changes in total community SA:V and bulk ratios of Si:C and Si:N can also be related to changes in community composition, which has been observed in the chronically Fe-limited HNLC waters of the subtropical Pacific Ocean and Southern Ocean [54]. For example, Fe-limitation could induce a shift toward smaller diatom species with a higher SA:V and more efficient Fe uptake relative to other cellular requirements [55–57]. Alternatively, an increase in the proportion of diatoms within the phytoplankton community could increase the total siliceous-based SA relative to total phytoplankton-based V, causing a similar increase in Si:C and Si:N. Based on our metatranscriptome analyses, we did not observe a significant difference in community composition between Fe treatments in this study. This could be due to the ability of bloom-forming diatoms to outcompete other phytoplankton groups for nutrients during upwelling events [29], or an ability to assimilate DFB-bound iron [58], albeit at far lower rates than unbound Fe. Irrespective of the mechanism, our observed increase in Si:C and Si:N in Fe-limited diatom communities seems unlikely to have been driven by modest changes in community composition as only *Thalassiosira* cells, which only contributed at most 13% of community composition at 72 and 120 h respectively, differed significantly in cell-specific Si production or in the degree of silicification when comparing among taxa (Fig. S6C, D). As neither taxon-specific SA:V of the dominant diatom genera, nor community composition differed between Fe-replete and Fe-limited treatments, we posit that the observed increase in Si:C and Si:N in Fe-limited diatoms was driven by cellular shifts in Si, C, and/or N content.

### Fe-limitation impacts on diatom Si, C, and N content

Previous studies within the CUZ have reported increased Si:chlorophyll and Si:C and preferential utilization of Si(OH)_4_ over NO_3_^−^ under Fe-limiting conditions [2]. These changes have been largely attributed to increases in cellular bSi content based on laboratory culture experiments demonstrating an increase in silica per cell under Fe-limiting conditions in some, but not all diatom species [13, 14, 59]. The prevailing mechanistic explanation is that Fe-limitation prolongs cell-cycle progression and, given the close association between silica production and the cell-cycle [15], allows more time for silica to be incorporated into the frustule [60]. However, evidence from both culture [10, 19, 44, 50] and field-based studies [6] suggest increased Si:C and Si:N in Fe-limited diatoms can also be due to reductions in cellular C and N content. The reliance of C and N assimilation on Fe-dependent proteins makes these processes particularly vulnerable to Fe-limitation. By decreasing the abundance of Fe-dependent proteins or substituting with an Fe-independent analog, cellular Fe quotas are reduced, but at the expense of C and N assimilation [23, 26, 61].

In our study, elevated Si:C and Si:N under Fe-limitation were driven primarily by decreases in cellular C and N without a concomitant increase in Si, proportionally similar to the plasticity exhibited by diatoms in previous findings [6, 12]. This is supported by decreases in both DIC and NO_3_^−^ uptake as well as declines in Chl *a* concentration. A significant reduction of transcript abundance for genes encoding the high Fe-requiring photosynthetic electron transport proteins *petC*, *petH*, *petJ*, as well as those encoding Fe-dependent nitrate and nitrite reductase proteins was also observed under Fe-limitation. No concomitant increase in cell-specific silica production rates or in the degree of silicification under Fe-limitation was observed in the dominant diatom genera. In fact, the only significant change in silica production rate was a nearly 50% decrease by the large morphotype of *Pseudo-nitzschia* that was largely driven by a decrease in the degree of silicification. We did measure a reduction in the newly formed frustule surface area of Fe-limited *Thalassiosira*, a possible indicator of a slower growth rate, but no significant change in silica production or degree of silicification was detected. Despite the impaired molecular C- and N-assimilation pathways and reduced C and N quota, the consistent bulk and single-cell bSi measurements between Fe treatments suggest Fe-limited diatoms were still able to sustain cell division rates comparable to Fe-replete cells. Furthermore, we observed that Fe limitation did not increase Si content using synchrotron X-ray fluorescence (SXRF) analysis of cellular elemental composition in two similar incubations conducted on the same cruise but at different locations (38° 39.30 N, 123°39.87 W and 42°40.00 N, 125°0.00 W, Twining, unpublished data). Previously, we demonstrated that SXRF- and PDMPO-based measurements of Si content provide comparable results [28]. At these two distinct sites, we found no difference in Si cell^−1^ or biovolume-normalized Si content (Si L^−1^) of *Chaetoceros* or *Pseudo-nitzschia* cells between Fe-replete and Fe-limited conditions (ibid).

### Decoupled silicon metabolism transcript abundance and silicification

In contrast to C and N metabolism, our incomplete understanding of Si metabolism has thus far hindered the identification of viable molecular markers for silica production or Si limitation. We explored two of the more highly conserved and better characterized proteins—SITs and Sin1 [47, 48, 62]—as candidates for markers of silica production and degree of silicification, respectively. Although other diatom-specific proteins involved in silica biomineralization have been well-characterized [63, 64], poor sequence conservation across diverse diatom species precludes their use as molecular markers [31].

Expression of clades A and B *SITs* has been reported to specifically increase in response to Fe-limitation [17], but our data identified higher abundance of clades C, D, and E *SITs* under Fe-limitation. At the Si(OH)_4_ concentrations typical of the CUZ (<30 µmol L^−1^ [65, 66]) active transport of Si should be facilitated by SITs [67] and closely linked to the rate of bSi production [68]. In our study, there was a general increase in *SIT* transcript abundance with Fe-limitation, but it was not consistent across *SIT* clades. For example, while clade C, D, and E *SIT* transcripts were significantly more abundant under Fe-limitation, those of clade A and clade B were not. Regardless, we did not observe a correlation between the changes in *Pseudo-nitzschia* cell-specific Si production and *SIT* transcript abundance at 72 h or between bulk Si uptake and *SIT* transcript abundance in *Thalassiosira, Chaetoceros*, and *Pseudo-nitzschia* at 120 h. This is not completely unexpected given findings of post-transcriptional and post-translational regulation of SITs [62, 69]. However, strong correlation between *SIT* protein abundance has been reported after prolonged (24 h) in Si-free media [66], thus it could be useful to explore, in targeted studies, whether *SIT* transcript abundance is diagnostic of severe Si-limitation in field samples.

The biomineralization of silica is aided by *Sin1*, a highly conserved diatom-specific protein localized to the silica deposition vesicle and crucial for proper valve formation [31, 32]. We hypothesized that alterations in valve formation or Si content by Fe-limitation could be driven by changes in expression of *Sin1*. However, we observed strikingly different expression patterns of *Sin1* among the diatoms investigated in this study. Despite a lack of increase in the degree of silicification, both *Thalassiosira* and *Chaetoceros* cells exhibited increases in *Sin1* transcript abundance at the later timepoint. In contrast, *Pseudo-nitzschia* exhibited no difference in *Sin1* abundance between Fe-replete and Fe-limited treatments even through there was a lower degree of silicification in the larger size class of Fe-limited *Pseudo-nitzschia*. The lack of changes in cell silica content in response to Fe limitation has been observed in previous studies and calls into question the physiological drivers of *SIT* and *Sin1* transcript abundance in Fe-limited *Thalassiosira, Chaetoceros*, and *Pseudo-nitzschia* [17, 18].

One possible explanation for Fe-limitation driven changes in *SIT* and *Sin1* expression without concomitant increases in bSi production or degree of silicification may stem from the close connection between Si(OH)_4_ uptake, silica production and the cell cycle. Studies in *T. pseudonana* have shown that *SITs* exhibit a diurnal pattern of expression [70, 71], and are expressed maximally during cell-cycle stages associated with peak silica production [62]. In addition, transcriptomic-based studies on *T. pseudonana* and *Phaeodactylum tricornutum* report *Sin1* expression is most highly correlated with other genes involved in cell division [70, 71]. In assemblages with low species diversity, such as in this study, intraspecific cell division can become partially entrained in the light:dark cycle [72]. Analyzing cell-cycle related transcripts within a single diatom species relies on the timing of cell-cycle progression being closely aligned (in phase) among the vast majority of cells present. While artificial upwelling and low species diversity may have initially entrained diatom cell cycles to the photocycle, the transcription of cell-cycle related genes and timing of cell-cycle progression [73] can be altered by limitation of nutrients such as Si, N, or Fe [15, 74, 75]. This may confound the interpretation of genus-specific transcription patterns of *SITs* and *Sin1* between Fe treatments if the cell cycles of each taxon were unsynchronized. While the C and N-assimilation genes discussed here have only been reported to respond to diel cycles [76], any potential cell-cycle control of C- and N- assimilation genes would similarly complicate interpretation. Further work disentangling the role of individual members of the SIT family as well as individual SIT clades may provide a better picture of the cellular and biogeochemical significance of SITs.

## Conclusions

In this study a diatom-dominated phytoplankton community presented characteristic signs of Fe-limitation including elevated cellular Si:C and Si:N ratios. Other studies reporting similar findings often invoke higher Si content as a mechanistic explanation, with implications for subsequent sinking and export given the ballasted nature of the silica-based cell wall. However, using single-cell, taxon-resolved measurements of the degree of diatom cell silicification, we found no difference in Si content between Fe-replete and Fe-limited cells, and instead found significant reductions in both C and N assimilation. Increased transcript abundance of the highly conserved *SIT* and *Sin1* genes did not correlate with rates of silicon uptake, cell-specific silica production, or with the degree of silicification, demonstrating that neither gene appear to be viable molecular markers for those respective processes within Fe-limited regimes. Our finding that none of the dominant diatom genera responded to Fe-limitation by thickening frustules complements previous field studies in both the Equatorial Pacific [6] and CUZ [28], lying in stark contrast to studies that suggest diatom frustule thickening is a common response to Fe-limitation [2]. Thus, evaluation of drivers of carbon export and opal burial in Fe-limited regimes must also consider mechanisms that do not depend on increased silica ballasting, such as increased aggregation [77], reduced grazing and associated respiratory losses [5], and viral infection [78].

## Methods

### Incubation setup

We conducted a deckboard multi-day incubation experiment at 35° 56.0710’N, 121° 44.0220’W within the California Upwelling Zone (CUZ) on board the *R/V* Melville (MV1405, Chief Scientist: Kenneth Bruland) from July 17–22, 2014. Details on incubation setup and downstream analysis are reported elsewhere [29, 30, 39]. Briefly, 200 L of water was collected from 96 m into acid-cleaned HDPE drums using an air-driven PTFE deck pump fitted with PTFE tubing. Water was distributed into eighteen 10 L acid-cleaned cubitainers using trace-metal clean techniques. Each cubitainer was amended with either 5 nmol L^−1^ FeCl_3_ (+Fe) or 200 nmol L^−1^ of the siderophore desferroxamine-B (+DFB), the latter to induce Fe limitation [79]. Unamended cubitainers served as controls (Ctrl). Cubitainers were incubated in plexiglass incubators shaded to ~33% incident irradiance and cooled with the flow-through seawater. At each timepoint, triplicate cubitainers were sacrificed at dawn for enumeration, dissolved and particulate analyses, cell-specific silica measurements, DIC and NO_3_^−^ uptake rate measurements, F_v_/F_m_, and RNA.

### RNA sequencing and analysis

RNA was extracted using an Ambion ToTALLY RNA Kit with a glass bead beating step, and one round of DNase 1 (Ambion) treatment [29, 41]. Samples at 120 h were sequenced in biological triplicate, but T0 and 72 h samples yielded low quantities of RNA necessitating the pooling of biological triplicates prior to library prep [29] using an Illumina TruSeq Stranded mRNA Library Prep and running on an Illumina HiSeq 2000. Trimmed reads were *de novo* assembled into contiguous sequences (contigs) with ABySS v.1.5.2 [80] and annotated using BLASTX+ v.2.2.21. Taxonomic annotation was assigned with MarineRefII database and functional annotation was assigned using Kyoto Encyclopedia of Genes and Genomes (KEGG) [81]. KEGG Ortholog (KO) classifications of diatom urea transporters, nitrite reductase, ammonium transporters were manually verified against known gene phylogenies and edited accordingly [82]. Iron Starvation Induced Proteins (ISIPs), silicon transporters (*SITs*), and silicanin-1 (*Sin1*) lack KO identifiers and were thus manually annotated using BLASTX+ v.2.5.0 [32, 48, 83]. To further classify *SIT* contigs into clades, using pplacer version 1.1.alpha19 with posterior probability calculated [84].

### Statistical analysis

Significance between treatments within a given timepoint was determined via one-way ANOVA using the R package *stats* v.3.6.2 followed by Tukey’s HSD multiple comparison test from *multcomp* v.1.4.13. When only two Fe treatments were available for comparison a Welch two-sample *t* test was used to calculate the level of significance. Community level differences in taxonomic contribution to mRNA reads were determined via PERMANOVA using the *adonis* function in the R package *vegan* v2.7. For transcript relative abundance data, normalization, differential abundance, and significance were analyzed within each taxonomic group using edgeR v.3.28.1 [85]. Significance between treatments at a given timepoint was determined using exactTest with tagwise dispersion and corrected for multiple testing using the Benjamini and Hochberg method, with a significance threshold of FDR < 0.05 [86].

## Supplementary information

Supplementary materials

Supplemental table 5

Supplemental table 6

Supplemental table 7

Supplemental table 8

Supplemental table 9

## Data Availability

All raw sequence data have been deposited in the NCBI sequence read archive under the accession no. SRP074302 (BioProject accession no. PRJNA320398) and SRP108216 (BioProject no. PRJNA388329). All data can be access through the Biological and Chemical Oceanography Data Management Office (Project number 559966, https://www.bco-dmo.org/deployment/559966).
