# Supplementary material for: Diminished carbon and nitrate assimilation drive changes in diatom elemental stoichiometry independent of silicification in an iron-limited assemblage
Source: ISME Commun. 2022 Jul 9;2:57. doi: 10.1038/s43705-022-00136-1 (PMC9723790; doi:10.1038/s43705-022-00136-1)
Supplement: Supplementary file 1 — Supplementary materials [file 43705_2022_136_MOESM1_ESM.pdf]

Supplemental Materials

Diminished carbon and nitrate assimilation drive changes in diatom elemental stoichiometry independent of silicification in an iron-limited assemblage

Michael A. Maniscalco[1], Mark A. Brzezinski[1], Robert H. Lampe[2], Natalie R. Cohen[3], Heather M. McNair[4], Kelsey A. Ellis[5], Matthew Brown[6], Claire P. Till[7], Benjamin S. Twining[8], Kenneth W. Bruland[9], Adrian Marchetti[5], Kimberlee Thamatrakoln[10]

[1] Marine Science Institute and The Department of Ecology Evolution and Marine Biology, University of California, Santa Barbara, CA 93106, USA
[2] Integrative Oceanography Division, Scripps Institution of Oceanography, University of California, San Diego, La Jolla, CA 92093, USA
[3] Skidaway Institute of Oceanography, University of Georgia, Savannah, GA 30602, USA
[4] University of Rhode Island, Graduate School of Oceanography, Narragansett, RI 02881, USA
[5] University of North Carolina, Chapel Hill, NC 27599, USA
[5] Flagler College, St. Augustine, FL 32084, USA
[7] Chemistry Department, California State Polytechnic University, Humboldt, Arcata, CA 95521, USA
[8] Bigelow Laboratory for Ocean Sciences, East Boothbay, ME 04544, USA
[9] Department of Ocean Sciences, University of California, Santa Cruz, CA 95064, USA
[10] Department of Marine and Coastal Sciences, Rutgers University, NJ 08901, USA

Corresponding authors
Kimberlee Thamatrakoln thamat@marine.rutgers.edu
Michael A. Maniscalco mmaniscalco@ucsb.edu

This PDF file includes:
Supplementary materials and methods
Table S1: Dissolved nutrient concentrations
Table S2: Particulate biomass concentrations
Table S3: Uptake rate values
Table S4: Ratios of particulate biomass concentrations
Figs. S1-S6
References for supplementary materials

Additional available files:
Table S5- Significance test results for biogeochemical measurements
Table S6- Community composition relative abundance data
Table S7- pplacer Most Recent Common Ancestor assignments
Table S8- Transcript abundance data from Fig. 3 and Fig. S3
Table S9- Confocal microscopy based PDMPO data from Fig. 4

Materials and methods

Dissolved and particulate analyses

Samples for dissolved nutrient analysis (NO_3^- + NO_2^-, PO_4, $Si(OH)_4$) were filtered through a GF/F filter and stored at 4°C until analysis on the ship using a Lachat 8000 Quick Chem Flow Injection Analysis system [1]. Concentrations of NO_3^- + NO_2^- are referred to simply as NO_3^- as concentrations of NO_2^- were negligible. Reference materials for nutrients in seawater (Lots BY and CA, KANSO Technos, Osaka, Japan) were used for quality control. Samples for biogenic Si were filtered onto 0.6 µm pore-size polycarbonate filters and stored at -20°C. Samples were digested shipboard with 0.2 N NaOH in Teflon tubes, and measured using the ammonium molybdate colorimetric assay [2]. For the initial (T0) dissolved Fe measurements the seawater samples were shipboard filtered and analyzed spectrophotometrically with ultraclean methods after [3]. All other dissolved Fe concentration samples were acidified and stored in acid-cleaned LDPE bottles for at least two months before preconcentration and analysis with a Thermo Fisher Element XR inductively coupled plasma mass spectrometer [4, 5].

To assess total particulate carbon (TPC), total particulate nitrogen (TPN), and nitrate (NO_3^-) uptake rates, 618 ml sub-samples of seawater from each cubitainer were each spiked with $Na_{15}NO_3$ at no more than 10 % of the ambient NO_3^-concentration and incubated deckboard for 8 h in a flow-through plexiglass incubator shaded to 33% incident irradiance. Following incubation, samples were gravity filtered through a 5 µm polycarbonate filter (47 mm) then rinsed with an artificial saline solution onto a precombusted 25 mm GF/F filter. Filters were stored at -20°C until analysis. Prior to analysis, filters were dried at 50°C for 24 h, then run on an elemental analyzer paired with an isotope ratio mass spectrometer (EA- IRMS). Uptake rates (ρ, NO_3^- per unit time) were calculated using a constant transport model (Eq. 3 from [6]). Biomass-specific NO_3^- uptake rates (V, NO_3^- per unit TPN per unit time) were also calculated according to the constant specific uptake model (Eq. 6; [6]). The reported values are considered conservative estimates or net uptake because $^{15}NO3$ uptake rates were not corrected for possible losses of ^{15}N in the form of dissolved organic nitrogen [7].

To assess dissolved inorganic carbon (DIC) uptake 60 ml sub-samples from each cubitainer were distributed into acid-cleaned light and dark bottles. Each bottle was spiked with 1.2 µCi of $NaH^{14}CO_3$. A 1 ml subsample was taken from each bottle and added to vials containing 6 mol L^{-1} NaOH to trap and validate the initial inorganic $H^{14}CO3$ quantities. The light and dark bottles were incubated for 6.5–8 h in a deckboard flow-through plexiglass incubator 33% incident irradiance. Each 60 mL sub-sample was filtered onto 5 µm polycarbonate filters. Blank control bottles containing 1.2 µCi of $NaH^{14}CO_3$ were filtered onto a GF/F filter and had counts similar to dark bottles. Filters were vacuumed dried, placed in scintillation vials with 0.5 ml of 6 mol L^{-1} HCl, degassed for 24 h and counted using a Beckman Coulter LS 6500 scintillation counter. Reported values are light bottles minus dark bottles. Biomass-specific DIC uptake rates (V_{DIC}) were calculated by normalizing DIC uptake to TPC.

Biomass-specific Si uptake measurements were calculated for samples at 72 h and 120 h by taking the natural log of the change in $Si(OH)_4$ concentration of a sample at a given time point (n) relative to the mean value of within the corresponding treatment at the previous time point (n-1) normalized to the mean bSi concentration of the corresponding treatment at the previous time point.

$$V_b = ln\left(\left([\mathrm{Si(OH)}_4]_n - [\,\overline{\mathrm{Si(OH)}_4}\,]_{n-1}\right) \times \left([\,\overline{\mathrm{bSi}}\,]_{n-1}\right)^{-1}\right) \text{ (Eq. 1)}$$

Cell specific silica measurements

Cell-specific and community bSi production rates were measured at 72 h using 244 nmol L^{-1} of using 2-(4-pyridyl)-5-((4-(2-dimethylaminoethylaminocarbamoyl)methoxy)phenyl)oxazole (PDMPO; LysoSensor Yellow/Blue DND-160, Molecular Probes) and incubated deckboard for 6 h prior to sampling.

From each PDMPO incubation, 50 mls was removed and centrifuged at 1,230 RCF for 10 min. Pellets were resuspended in 10 mL of methanol and stored for >24 h at 4ºC to remove unbound PDMPO and photopigments. An aliquot (targeting 2-4 x 10^4 cells) was removed and 10 ml of MilliQ water was added to the remaining sample to remove precipitated salts and centrifuged (1,230 RCF, 10 min). All but 200 µL of supernatant was removed. Cells were then pipetted onto a polylysine-coated glass slide and left to dry. A #1.5 cover slip was adhered with ProLong Gold Antifade (Life Technologies), sealed with nail polish, and stored in the dark at 4°C [8–10].

PDMPO-labeled cells were imaged using an Olympus Fluoview 1000 Spectral Confocal microscope and analyzed as described [8–10]. In brief, cells were serially imaged in discrete 420 nm depths in short (PDMPO, ex 405/em 450-550 nm) and long (autofluorescence from photopigments, ex 559/em 617-718 nm) wavelengths to allow quantification of both the volume and surface area of newly produced silica, as well as total fluorescence. Images were assembled into 3D renderings (voxels) by digitally extrapolating between depths and removing photopigment fluorescence, ensuring only the PDMPO fluorescence was used in silica quantification. PDMPO fluorescence was converted into a mass of bSi using a standard curve generated from laboratory-grown fully labelled diatom cells of known silica content [8]. The degree of cellular silicification (fmol Si μm^{-2}), or frustule thickness, was calculated by dividing the rate of silica production (pmol Si $cell^{-1}$ d^{-1}) by the rate of new surface area produced (μm^2 $cell^{-1}$ d^{-1}) :

$$Degree\ of\ silicification = \frac{Si\ production}{new\ frustule\ SA} \qquad \text{(Eq. 2)}$$

The valve apical length and pervalvar width were used to calculate the total volume and surface area using formulae for a cylinder for *Thalassiosira and Chaetoceros.* Valve apical length and transapical width were used to calculate total volume and surface area using formulae for a prolate spheroid for *Pseudo-nitzschia*.

RNA extraction and metatranscriptome analysis

Samples for metatranscriptomic analysis were filtered onto 142 mm, 0.8 µm pore-size Pall Supor filters, flash frozen, and stored at -80º C. RNA was extracted using an Ambion ToTALLY RNA Kit with a glass bead beating step, and one round of DNase 1 (Ambion) treatment [11, 12]. Samples from the first time point (72 h) yielded low quantities of RNA necessitating the pooling of biological triplicates prior to library prep [12] using an Illumina TruSeq Stranded mRNA Library Prep kit and HiSeq v4 reagents. Samples were barcoded and run on an Illumina HiSeq 2000 (125bp, paired-end).

Reads from each site were trimmed for quality and removal of adapters using Trimmomatic v0.32 (paired-end mode, adaptive quality trim with 40 bp target length and strictness of 0.6, minimum length of 36 bp) [13]. Overlapping trimmed paired reads were interleaved into single reads with BBMerge v8.0. Merged pairs and non-overlapping paired-end reads were assembled into contigs using ABySS v1.5.2 with varied k-mer sizes [14]. The different k-mer size assemblies were merged using Trans-ABySS v1.5.3 to remove redundant contigs and contigs

<120 bp [15]. Assemblies from all sites were merged with Trans-ABySS and duplicates further removed with GenomeTools v1.5.1 [16]. Read counts were estimated using the quasi-mapping method implemented in Salmon v0.73-beta with the seqBias option [17].

NCBI taxonomic IDs were assigned to contigs by best homology using BLASTX v2.2.31 (E-value $\leq 10^{-3}$) with MarineRefII database. Taxonomic information was mapped to the NCBI taxonomic IDs of each contig using the National Center for Biotechnology Information's (NCBI) Taxonomy Database [18], and manually curated) to ensure proper assignment and use of common phytoplankton taxonomic ranks (https://github.com/marchettilab/metatranscriptomicsPipeline. Gene function was assigned using BLASTX (E-value $\leq 10^{-3}$) with the Kyoto Encyclopedia of Genes and Genomes (KEGG; Release 75) [19]. For analysis of KEGG Orthologs (KO), the top hit with a KO number from the top 10 hits was chosen [11, 20]. KO classifications of diatom urea transporters, nitrite reductase, ammonium transporters were manually verified against known gene phylogenies and edited accordingly [21].

Iron Starvation Induced Proteins (ISIPs), silicon transporters (*SITs*), and silicanin-1 (*Sin1*) lack KO identifiers and were thus manually annotated using BLAST [22–24]. To further classify *SIT* sequences by clade, a maximum-likelihood tree was constructed from a reference alignment [24] using RAxML version 8.2.12 with the PROTGAMMAWAGF substitution model and 100 bootstraps [25]. Amino acid sequences corresponding to *SIT* contigs were aligned to the reference alignment using hmmalign and the HMM profile created from the reference alignment (built using hmmbuild 3.2.1). Associated alignment and tree files were packaged using taxtastic version 0.8.3. *SIT* contigs were placed on the reference tree using pplacer version 1.1.alpha19 with posterior probability calculated [26]. The most closely related reference sequence was assigned to each *SIT* contig using guppy version 1.1.alpha19. *SIT* clade assignments were included as KO identifiers for downstream analysis.

Prior to differential expression analysis, raw counts were aggregated within diatom genera by KO number. For genes lacking a KO number, e.g. *SITs*, ISIPs, Sin-1, etc., aggregation was done based on gene assignment through KEGG annotation or BLASTX query of supplemental databases. Prior to differential expression analysis, raw counts were aggregated within diatom genera by KO number. For genes lacking a KO number, e.g. SITs, ISIPs, Sin-1, etc., aggregation was done based on gene assignment through KEGG annotation or BLASTX query of supplemental databases. Genus-specific aggregation of functionally annotated reads reduces redundancy and allows the use of tools originally designed for single organism RNAseq analysis (e.g. DESeq2, edgeR). This is necessary for microbial community transcriptomic analysis due to methodological and computational difficulties in resolving species-level, differential transcript expression (Toseland et al. 2014; Alexander et al. 2015; Kopf et al. 2015; Cohen et al. 2017b, 2021; Hu et al. 2018; Lampe et al. 2018b). However, this approach does not resolve species-level contig expression, nor does it *a priori* resolve clade-specific expression patterns for genes that belong to multigene families. In the case of SITs, individual clades were identified through phylogenetic analysis as previously done (Durkin et al. 2012, 2016), and manually annotated to allow interrogation of SIT transcripts at the clade level. Additionally, annotations for urea transporters 1 and 2 were grouped together because of there close phylogenic relationship and shared expression pattern in response to nitrogen supply (Smith et al. 2019). A similar approach for other multigene families, such as the ammonium transporters (AMTs) and nitrate transporters (NRT2s) was not used because 1) there are no studies reporting a clade-specific response to Fe limitation and 2) expression of these genes in response to nitrogen supply does not appear to be clade-specific (Smith et al. 2019), thus interrogating clade-

level expression patterns of these genes in our metatranscriptomes would be unlikely to yield interpretable results.

Phytoplankton analysis

Cell abundance and community composition were determined using microscopy [33]. Whole seawater was preserved in 2% Lugols and settled for > 24 h in an Utermöhl chamber. At least 400 cells were counted in at least five fields of view at 100×, 200×, and 400× magnification using a Leica DMIL inverted microscope.

The maximum photochemical quantum efficiency of PSII (F_v/F_m) of dark adapted cells was calculated from fluorescence induction measurements using a Satlantic FIRe as described [34–36] and blank corrected with 0.2 µm filtered seawater.

Supplemental tables

Table S1: Dissolved macronutrient concentrations of initial upwelled water and control (Ctrl), Fe addition (+Fe), and DFB addition (+DFB) treatments at 72 and 120 h (n = 3, except for dFe concentration within the T0 saple where n = 1 and the Ctrl and +DFB treatments at 120h where n = 2).

	Silicic acid (µmol L^{-1})		Nitrate (µmol L^{-1})		Ortho-phosphate (µmol L^{-1})		Dissolved iron (nmol L^{-1})	
	Mean	St. dev	Mean	St. dev	Mean	St. dev	Mean	St. dev
T0	21.69	0.12	21.86	0.22	1.76	0.04	0.82	NA
Ctrl 72h	19.81	0.07	20.34	0.10	1.67	0.02	0.57	0.20
+Fe 72h	19.83	0.21	20.45	0.12	1.68	0.01	1.82	0.71
+DFB 72h	20.16	0.72	21.03	0.64	1.73	0.08	1.33	0.18
Ctrl 120h	14.70	2.87	15.07	2.75	1.37	0.27	0.40	0.10
+Fe 120h	16.37	1.16	15.30	1.41	1.35	0.10	1.09	0.47
+DFB 120h	15.91	0.66	17.53	0.86	1.54	0.12	1.43	0.59

Table S2: Particulate biomass concentrations of initial upwelled water and control (Ctrl), Fe addition (+Fe), and DFB addition (+DFB) treatments at 72 and 120 h (n = 3).

	TPC > 5 µm (µmol L^{-1})		TPN > 5 µm (µmol L^{-1})		bSi > 1.2 µm (µmol L^{-1})		Chl a > 5 µm (µg L^{-1})	
	Mean	St. dev	Mean	St. dev	Mean	St. dev	Mean	St. dev
T0	2.90	0.46	0.10	0.03	0.19	0.00	0.05	0.01
Ctrl 72h	6.04	2.32	0.96	0.82	0.33	0.05	0.53	0.04
+Fe 72h	6.22	1.31	1.27	0.65	0.37	0.04	0.69	0.12
+DFB 72h	4.30	1.63	0.34	0.10	0.34	0.10	0.22	0.03
Ctrl 120h	49.86	24.50	6.64	3.41	4.68	2.37	6.76	2.97
+Fe 120h	59.73	8.02	7.79	1.15	4.22	1.16	9.82	1.75
+DFB 120h	24.60	3.81	2.97	0.51	3.95	0.64	3.18	0.47

Table S3: The values of dissolved inorganic carbon, nitrate, and dissolved silicon uptake rates of initial upwelled water and control (Ctrl), Fe addition (+Fe), and DFB addition (+DFB) treatments at 72 and 120 h (n = 3). These data are presented in Fig. 1D.

	V_{DIC} (d^{-1})		V_N (d^{-1})		V_{Si} (d^{-1})	
	Mean	St. dev	Mean	St. dev	Mean	St. dev
T0	0.003	0.0006	0.06	0.02	NA	NA
Ctrl 72h	0.140	0.0700	0.54	0.25	0.79	0.01
+Fe 72h	0.210	0.0700	0.58	0.33	0.79	0.04
+DFB 72h	0.050	0.0200	0.40	0.04	0.70	0.17
Ctrl 120h	0.420	0.1100	1.32	0.03	1.30	0.37
+Fe 120h	0.510	0.0700	1.57	0.09	1.10	0.16
+DFB 120h	0.140	0.1300	0.79	0.02	1.25	0.08

Table S4: The values of the ratios of particulate biomass concentrations of initial upwelled water and control (Ctrl), Fe addition (+Fe), and DFB addition (+DFB) treatments at 72 and 120 h (n = 3). These data are presented in Fig. 1C. The mean Redfield (1963) proportions are provided in the bottom row as a reference point.

	TPC:TPN		bSi:TPC		bSi:TPN	
	Mean	St. dev	Mean	St. dev	Mean	St. dev
T0	31.48	5.49	3.35e-02	5.31e-03	2.13	0.61
Ctrl 72h	9.48	5.81	2.99e-02	1.13e-02	0.52	0.35
+Fe 72h	5.92	2.96	3.11e-02	9.01e-03	0.36	0.20
+DFB 72h	13.11	4.88	4.54e-02	2.34e-02	1.05	0.33
Ctrl 120h	7.59	0.27	4.67e-02	1.07e-03	0.71	0.01
+Fe 120h	7.68	0.12	3.49e-02	4.79e-03	0.54	0.07
+DFB 120h	8.31	0.51	8.02e-02	2.97e-03	1.33	0.03
Redfield	6.63	NA	1.51e-01	NA	1.10	NA

Figures

Figure S1: Macronutrients concentrations. Concentrations of nitrate (NO_3, black), phosphate (PO_4, blue), and silicic acid ($Si(OH)_4$, red) of the initial water mass (T0) and control (Ctrl), iron (+Fe) and DFB addition (+DFB) treatments at 72 and 120 h.

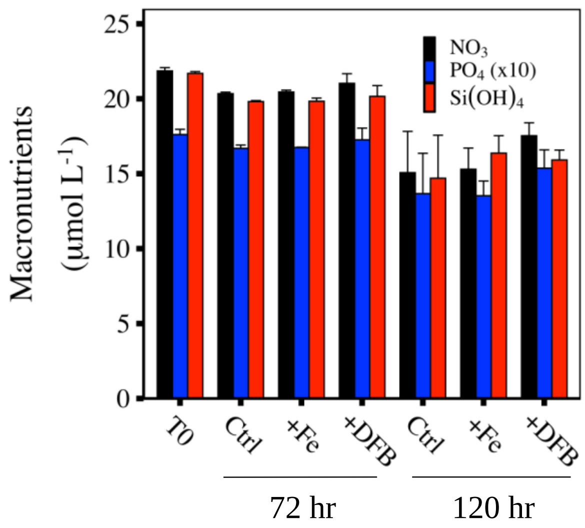

Figure S2: Percent relative abundance of diatom genera. *Chaetoceros* (black), *Pseudo-nitzschia* (gray), and *Thalassiosira* (black) percent relative abundance within diatom community in the initial water mass (T0) and control (Ctrl), iron (+Fe) and DFB addition (+DFB) treatments at 72 and 120 h based on taxonomically annotated mRNA reads.

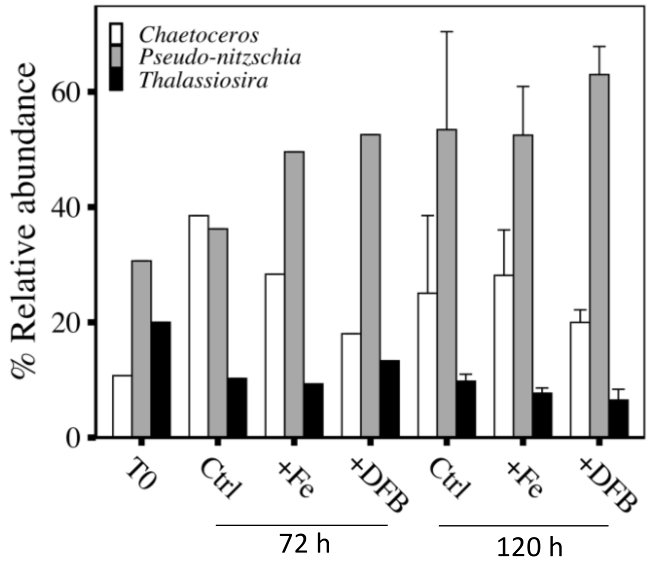

Figure S3: Molecular indicators of Fe limitation in the dominant diatom genera. Transcript abundance for iron starvation induced Protein 3 (ISIP3) in (A) *Thalassiosira* and (B) *Chaetoceros*. (C) For *Pseudo-nitzschia*, the iron limitation index (*Pseudo-nitzschia* ILI) was calculated (see Methods) with positive values >0.5 indicative of iron stress and negative values <-0.5 indicative of Fe replete growth (both thresholds indicated by the dashed lines). Statistical test results and significance are shown in Table S2.

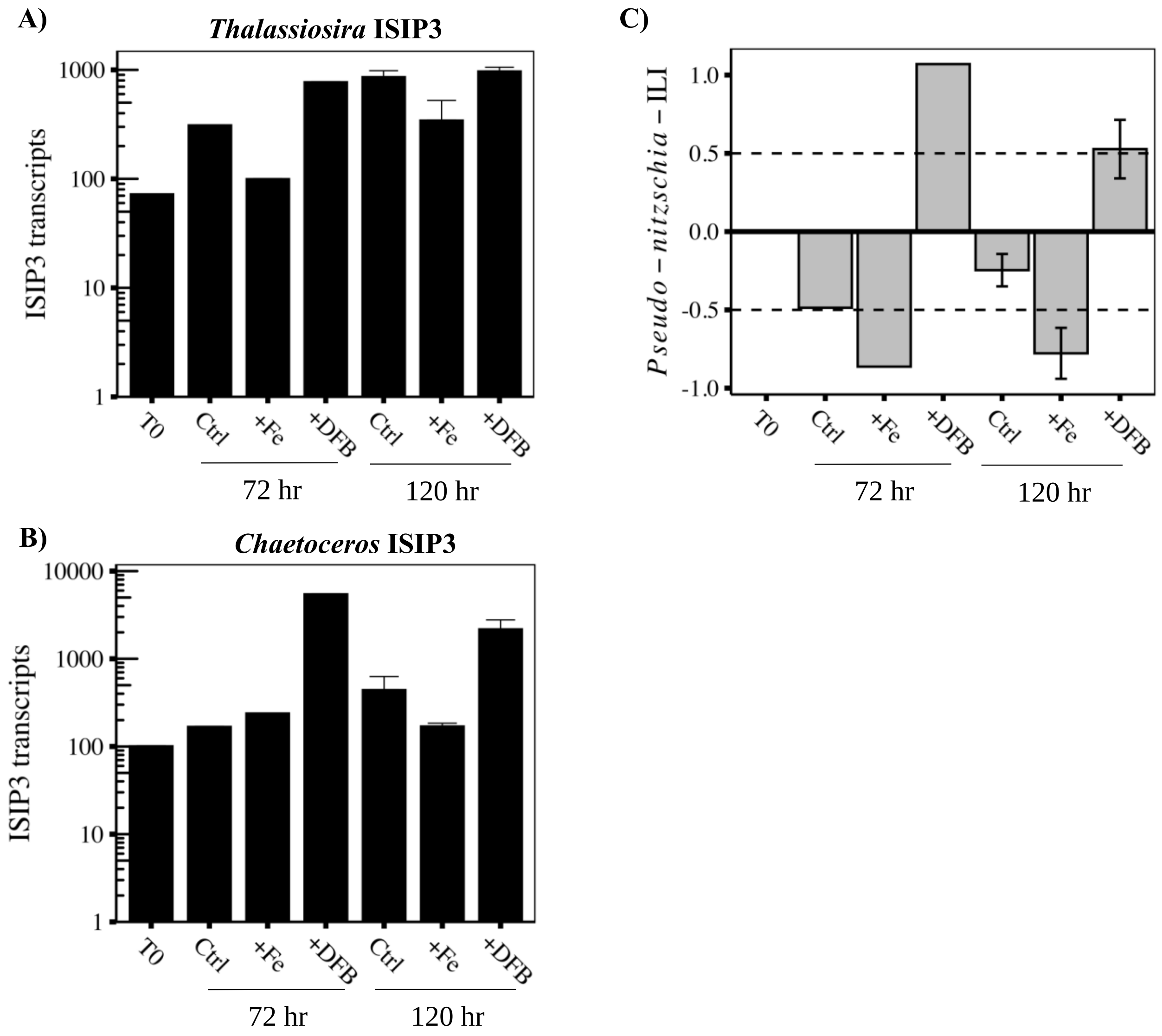

Figure S4: Silicon transporter phylogenetic tree. Phylogenetic reference tree containing 451 SIT amino acid sequences based on the reference alignment provided by Durkin et al. [24] and calculated using RAxML. Triangular nodes collapse closely related sequences together with the magnitude of the triangle proportional to the number of sequences present in the node. The tree is rooted with a clade containing SIT sequences encoded only by non-diatom organisms and labeled as “outgroup”. Bootstrap values >50 are shown (100 replicates).

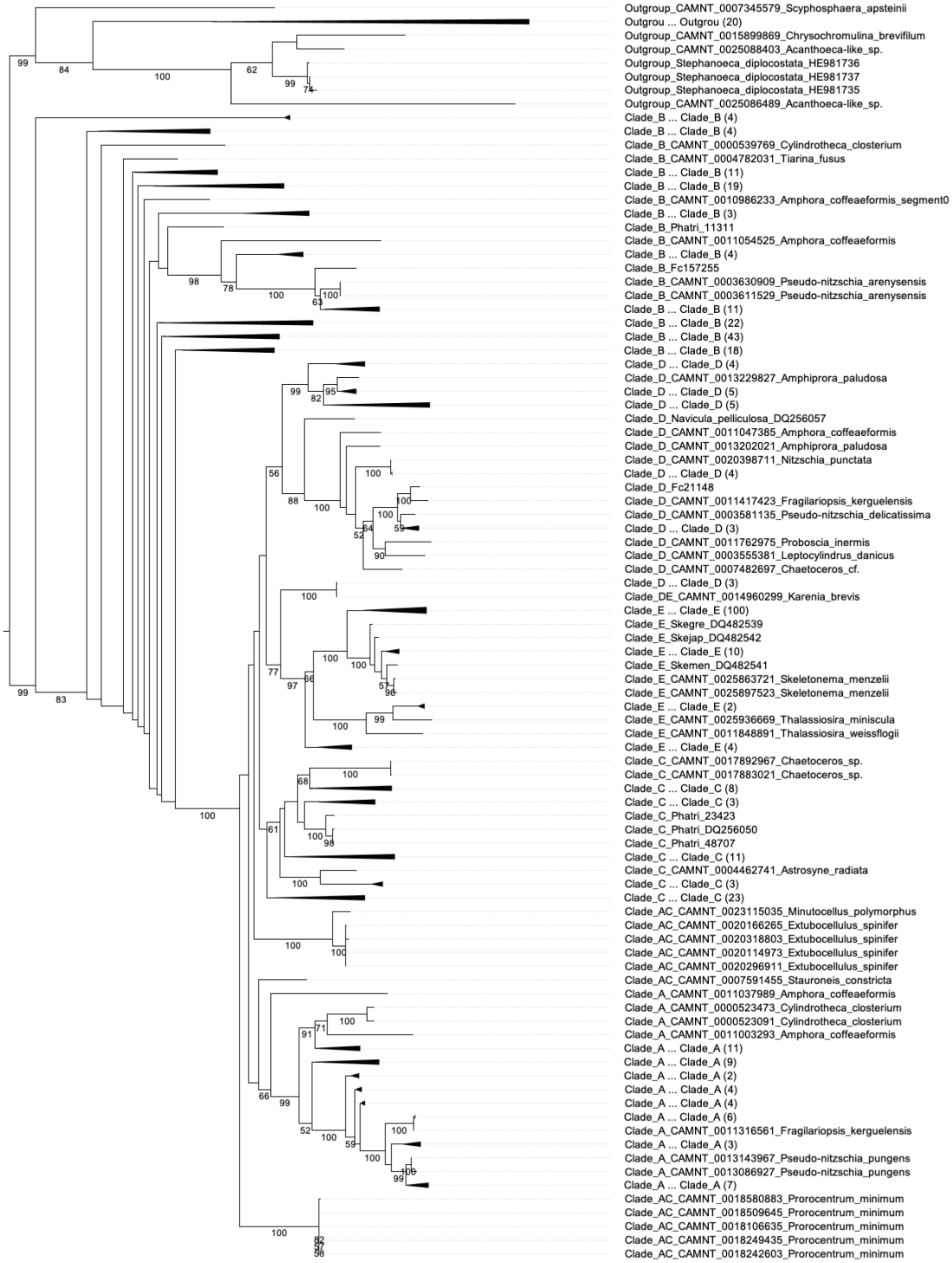

Figure S5: Confocal and 3D reconstruction images of diatom cells stained with PDMPO. PDMPO (ex 405/em 450-550 nm) is displayed in blue (panels A, D, and G), chlorophyll autofluorescence (ex 559 nm/ em 617-718 nm) is displayed in red/orange (panels B, E, and H), and a 3D reconstruction. The three diatom genera imaged are (A, B, C) a centric *Coscinodiscus*-like cell, (D, E, F), a short chain of *Chaetoceros* cells, and (G, H, I) a short chain of the ~90 µm *Pseudo-nitzschia* cells.

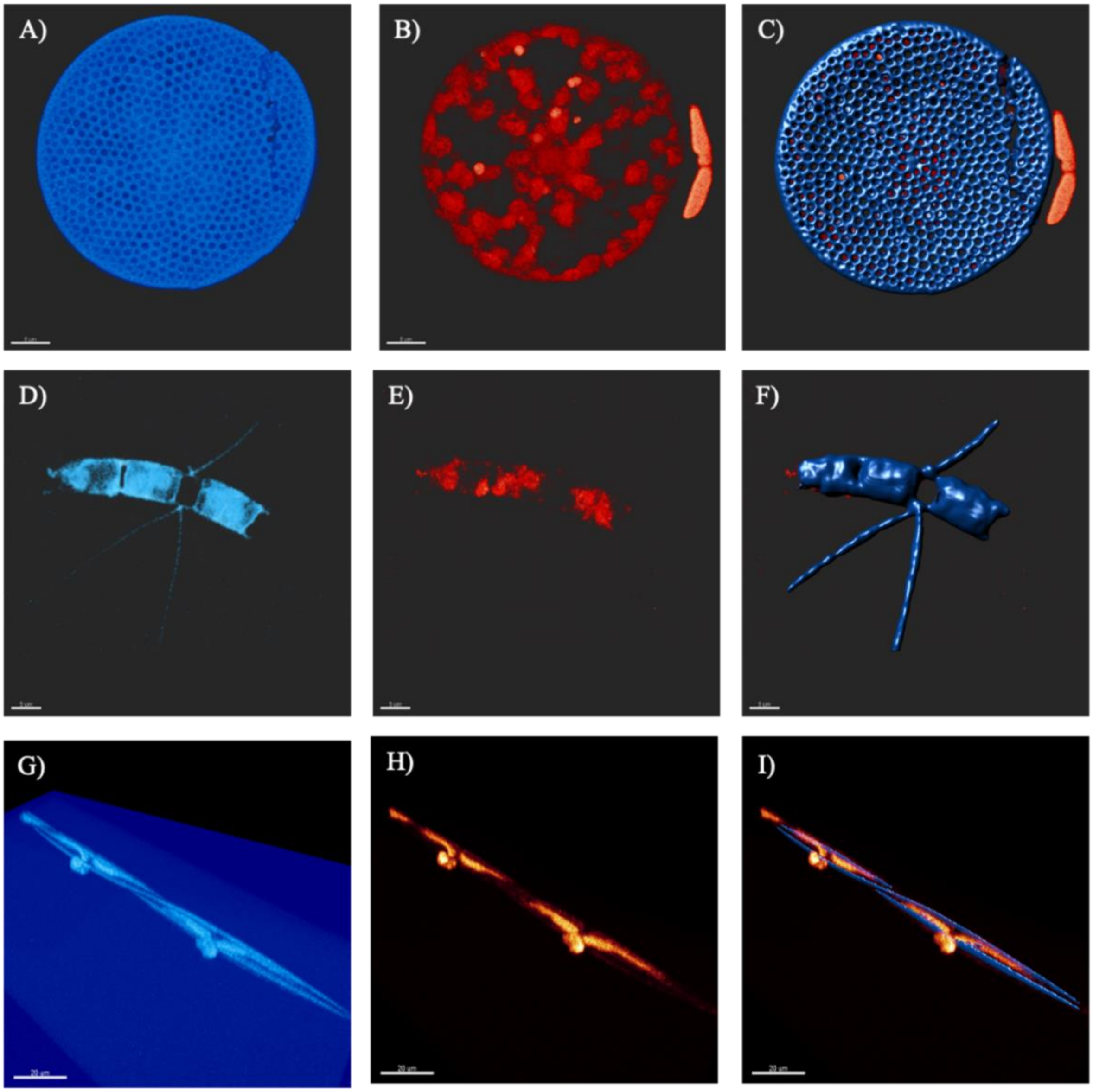

Figure S6: Morphological characteristics of diatom cells from incubations. Box and whiskerplots of (A) length normalized width measurements in *Pseudo-nitzschia* (45 and 90 µm size classes), (B) surface area to volume ratio (SA:V) for *Thalassiosira*, *Chaetoceros*, and *Pseudo-nitzschia* (45 and 90 µm size class) within the control (Ctrl; dark grey), +Fe (light grey), and +DFB (white) treatments at 72 h, (C) bSi production (pmol Si cell^{-1} d^{-1}, and (D) degree of silicification (fmol Si µm^{-2}). The center line represents the median and the boxes display the upper and lower quartiles with whiskers extending 1.5 times the interquartile range. Open diamonds represent the mean. Black dots denote data from individual cells. Samples with <5 cells were excluded and are denoted "nd". Significance was determined within each taxonomic group for A) and B) and between taxonomic groups for C) and D) by Welch two-sample t-test when comparing between two samples or one-way ANOVA and Tukey's HSD post hoc test when comparing among three samples. Lowercase letters delineate the statistically significant different groups (p < 0.05). The vertical axes are plotted at a logarithmic ($\log_{10}$) scale. Statistical test results and significance are shown in Table S5.

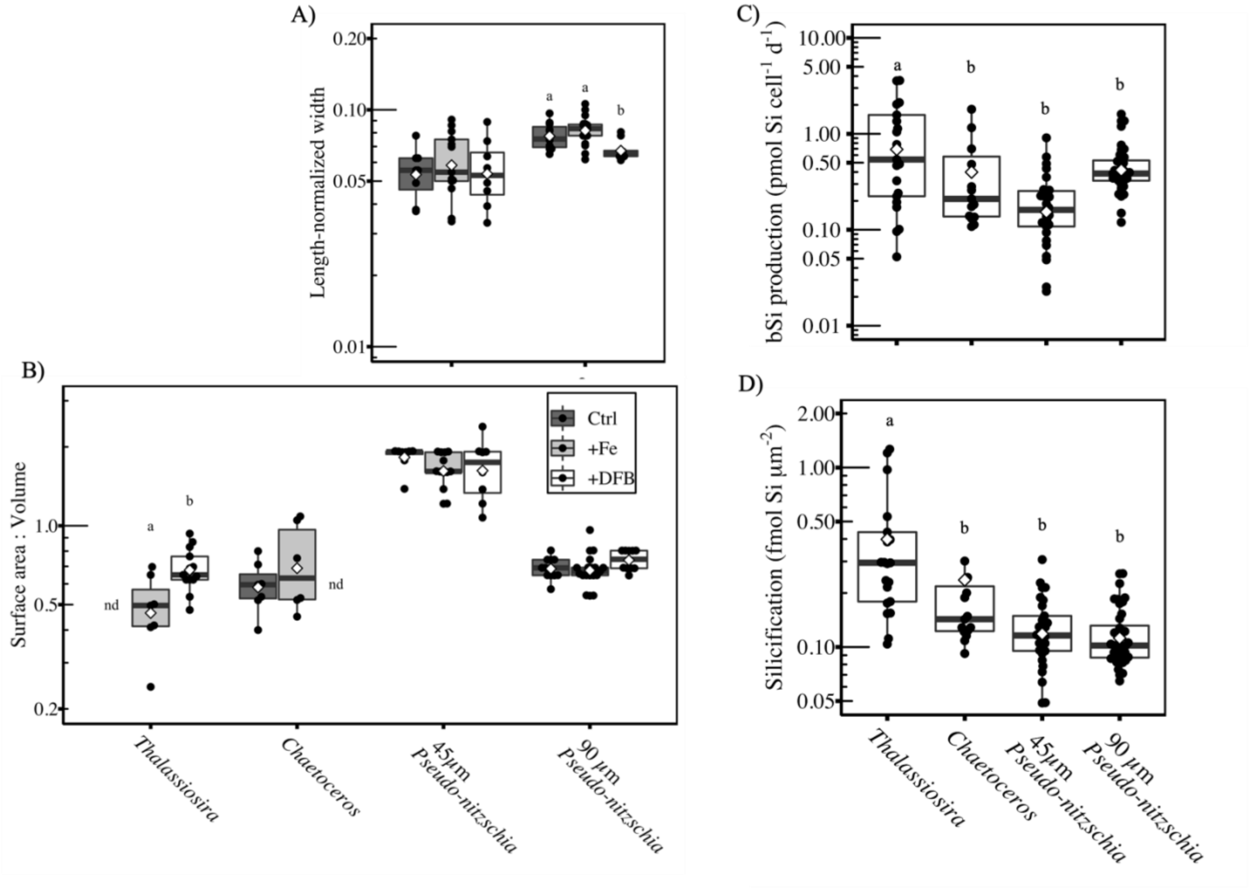

References

1. Parsons TR, Maita Y, Lalli CM. A manual of chemical and biological methods for seawater analysis. 1984. Pergamon Press.
2. Krause JW, Nelson DM, Lomas MW. Biogeochemical responses to late-winter storms in the Sargasso Sea, II: Increased rates of biogenic silica production and export. *Deep Res Part I Oceanogr Res Pap* 2009; **56**: 861–874.
3. Biller D V., Coale TH, Till RC, Smith GJ, Bruland KW. Coastal iron and nitrate distributions during the spring and summer upwelling season in the central California Current upwelling regime. *Cont Shelf Res* 2013; **66**: 58–72.
4. Parker CE, Brown MT, Bruland KW. Scandium in the open ocean: A comparison with other group 3 trivalent metals. *Geophys Res Lett* 2016; **43**: 2758–2764.
5. Biller D V., Bruland KW. Sources and distributions of Mn, Fe, Co, Ni, Cu, Zn, and Cd relative to macronutrients along the central California coast during the spring and summer upwelling season. *Mar Chem* 2013; **155**: 50–70.
6. Dugdale RC, Wilkerson FP. The use of 15N to measure nitrogen uptake in eutrophic oceans; experimental considerations,. *Limnol Oceanogr* 1986; **31**: 673–689.
7. Bronk DA, Gilbert PM, Ward BB. Nitrogen Uptake, Dissolved Organic Nitrogen Release, and New Production. *Science (80-)* 1994; **265**: 1843–1846.
8. McNair HM, Brzezinski MA, Krause JW. Quantifying diatom silicification with the fluorescent dye, PDMPO. *Limnol Oceanogr Methods* 2015; **13**: 587–599.
9. McNair HM, Brzezinski MA, Till CP, Krause JW. Taxon-specific contributions to silica production in natural diatom assemblages. *Limnol Oceanogr* 2018; **63**: 1056–1075.
10. McNair HM, Brzezinski MA, Krause JW. Diatom populations in an upwelling environment decrease silica content to avoid growth limitation. *Environ Microbiol* 2018; **20**: 4184–4193.
11. Cohen NR, Ellis KA, Lampe RH, McNair HM, Twining BS, Maldonado MT, et al. Diatom Transcriptional and Physiological Responses to Changes in Iron Bioavailability across Ocean Provinces. *Front Mar Sci* 2017; **4**: 1–20.
12. Lampe RH, Cohen NR, Ellis KA, Bruland KW, Maldonado MT, Peterson TD, et al. Divergent gene expression among phytoplankton taxa in response to upwelling. *Environ Microbiol* 2018; **20**: 3069–3082.
13. Bolger AM, Lohse M, Usadel B. Trimmomatic: A flexible trimmer for Illumina sequence data. *Bioinformatics* 2014; **30**: 2114–2120.
14. Birol I, Jackman SD, Nielsen CB, Qian JQ, Varhol R, Stazyk G, et al. De novo transcriptome assembly with ABySS. *Bioinformatics* 2009; **25**: 2872–2877.
15. Robertson G, Schein J, Chiu R, Corbett R, Field M, Jackman SD, et al. De novo assembly and analysis of RNA-seq data. *Nat Methods* 2010; **7**: 909–912.
16. Gremme G, Steinbiss S, Kurtz S. Genome tools: A comprehensive software library for efficient processing of structured genome annotations. *IEEE/ACM Trans Comput Biol Bioinforma* 2013; **10**: 645–656.
17. Patro R, Duggal G, Love MI, Irizarry RA, Kingsford C. Salmon provides fast and bias-aware quantification of transcript expression. *Nat Methods* 2017; **14**: 417–419.
18. Keeling PJ, Burki F, Wilcox HM, Allam B, Allen EE, Amaral-Zettler LA, et al. The Marine Microbial Eukaryote Transcriptome Sequencing Project (MMETSP): Illuminating the Functional Diversity of Eukaryotic Life in the Oceans through Transcriptome Sequencing. *PLoS Biol* 2014; **12**.
19. Kanehisa M, Furumichi M, Tanabe M, Sato Y, Morishima K. KEGG: new perspectives on genomes, pathways, diseases and drugs. *Nucleic Acids Res* 2017; **45**: D353--D361.

20. Lampe RH, Mann EL, Cohen NR, Till CP, Thamatrakoln K, Brzezinski MA, et al. Different iron storage strategies among bloom-forming diatoms. *Proc Natl Acad Sci* 2018; **115**: E12275–E12284.
21. Smith SR, Dupont CL, McCarthy JK, Broddrick JT, Oborník M, Horák A, et al. Evolution and regulation of nitrogen flux through compartmentalized metabolic networks in a marine diatom. *Nat Commun* 2019; **10**: 4552.
22. Morrissey J, Sutak R, Paz-Yepes J, Tanaka A, Moustafa A, Veluchamy A, et al. A novel protein, ubiquitous in marine phytoplankton, concentrates iron at the cell surface and facilitates uptake. *Curr Biol* 2015; **25**: 364–371.
23. Kotzsch A, Gröger P, Pawolski D, Bomans PHH, Sommerdijk NAJM, Schlierf M, et al. Silicanin-1 is a conserved diatom membrane protein involved in silica biomineralization. *BMC Biol* 2017; **15**: 9–11.
24. Durkin CA, Koester JA, Bender SJ, Armbrust VE. The evolution of silicon transporters in diatoms. *J Phycol* 2016; **52**: 716–731.
25. Stamatakis A. RAxML version 8: A tool for phylogenetic analysis and post-analysis of large phylogenies. *Bioinformatics* 2014; **30**: 1312–1313.
26. Matsen FA, Kodner RB, Armbrust VE. pplacer: linear time maximum-likelihood and Bayesian phylogenetic placement of sequences onto a fixed reference tree. *BMC Bioinformatics* 2010; **11**: 538.
27. Cohen NR, McIlvin MR, Moran DM, Held NA, Saunders JK, Hawco NJ, et al. Dinoflagellates alter their carbon and nutrient metabolic strategies across environmental gradients in the central Pacific Ocean. *Nat Microbiol* 2021; **6**: 173–186.
28. Alexander H, Rouco M, Haley ST, Wilson ST, Karl DM, Dyhrman ST. Functional group-specific traits drive phytoplankton dynamics in the oligotrophic ocean. *Proc Natl Acad Sci U S A* 2015; **112**: E5972–E5979.
29. Hu SK, Liu Z, Alexander H, Campbell V, Connell PE, Dyhrman ST, et al. Shifting metabolic priorities among key protistan taxa within and below the euphotic zone. *Environ Microbiol* 2018; **20**: 2865–2879.
30. Toseland A, Moxon S, Mock T, Moulton V. Metatranscriptomes from diverse microbial communities: Assessment of data reduction techniques for rigorous annotation. *BMC Genomics* 2014; **15**: 1–7.
31. Kopf A, Kostadinov I, Wichels A, Quast C, Glöckner FO. Metatranscriptome of marine bacterioplankton during winter time in the North Sea assessed by total RNA sequencing. *Mar Genomics* 2015; **19**: 45–46.
32. Durkin CA, Marchetti A, Bender SJ, Truong T, Morales RL, Mock T, et al. Frustule-related gene transcription and the influence of diatom community composition on silica precipitation in an iron-limited environment. *Limnol Oceanogr* 2012; **57**: 1619–1633.
33. Utermöhl H. Methods of collecting plankton for various purposes are discussed. *SIL Commun 1953-1996* 1958; **9**: 1–38.
34. Falkowski PG, Kiefer DA. Chlorophyll-A fluorescence in phytoplankton - relationship to photosynthesis and biomass. *J Plankton Res* 1985; **7**: 715–731.
35. Kolber ZS, Prasil O, Falkowski PG. Measurements of variable chlorophyll fluorescence using fast repetition rate techniques: defining methodology and experimental protocols. *Biochim Biophys ACTA-BIOENERGETICS* 1998; **1367**: 88–106.
36. Gorbunov MY, Falkowski PG. Fluorescence induction and relaxation (FIRe) technique and instrumentation for monitoring photosynthetic processes and primary production in aquatic ecosystems. *Photosynth Fundam Asp to Glob Perspect 13th Int Congr Photosynth* 2004; 1029–1031.
